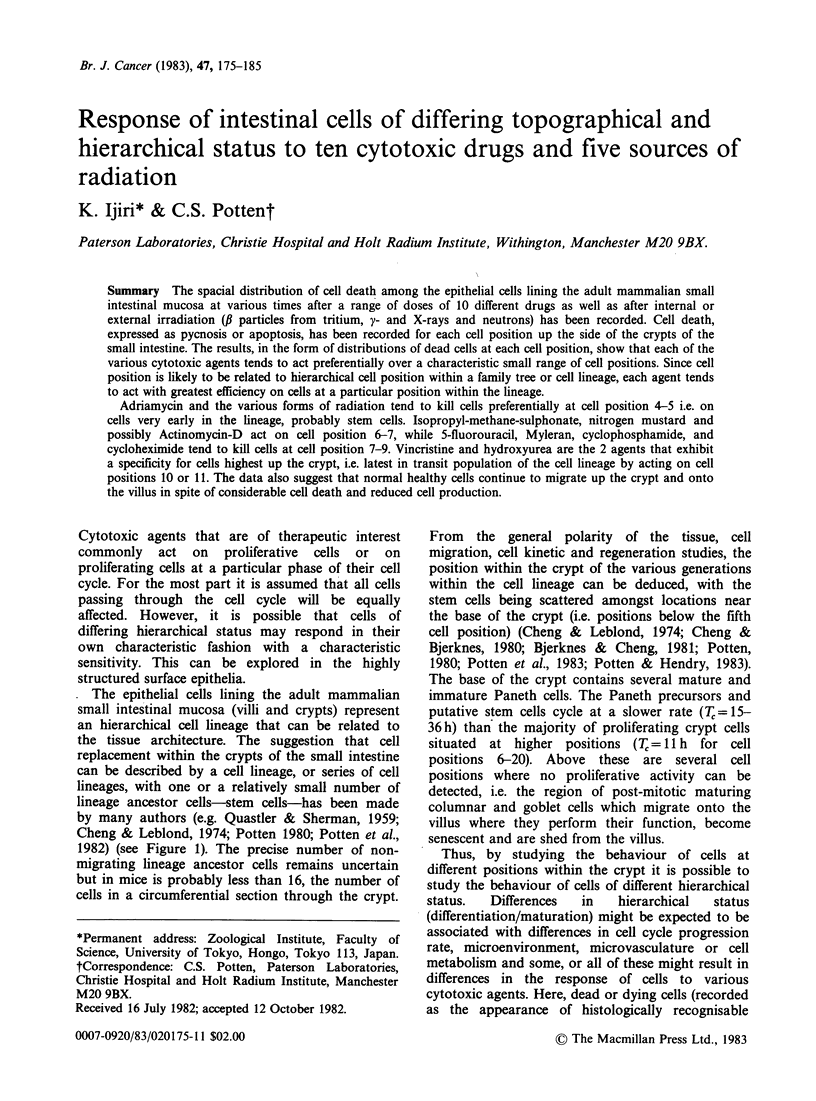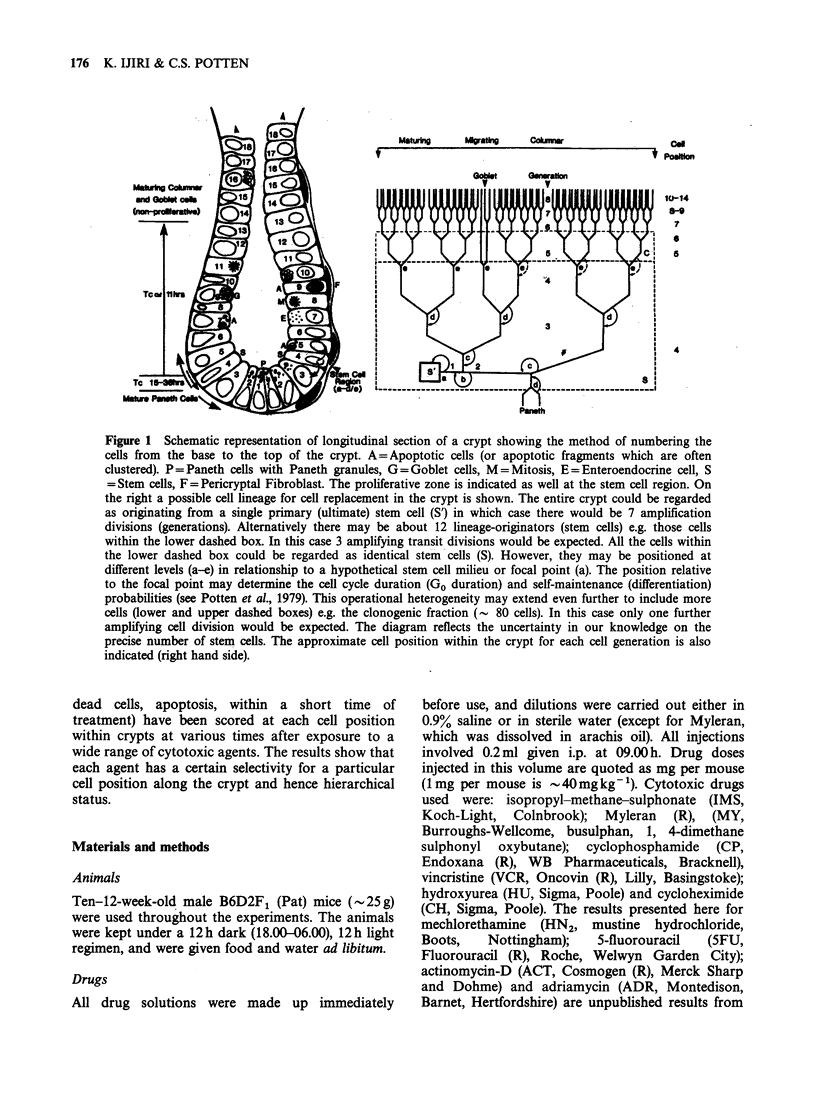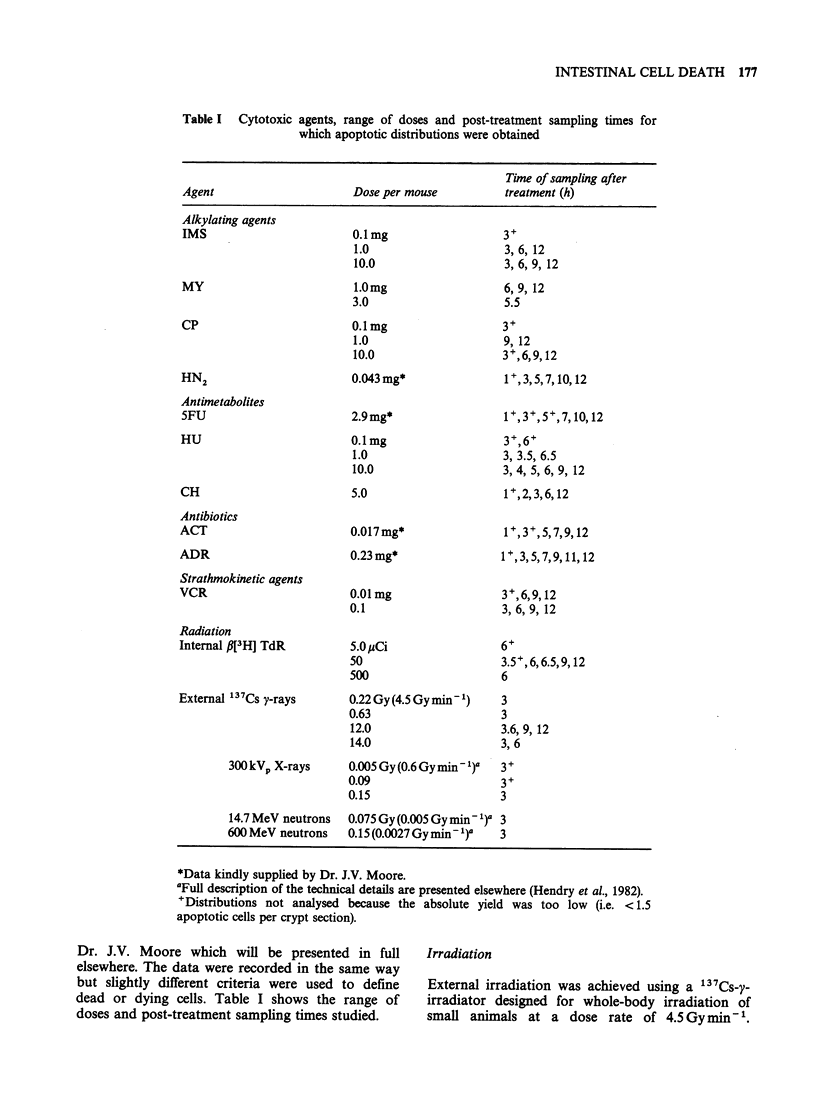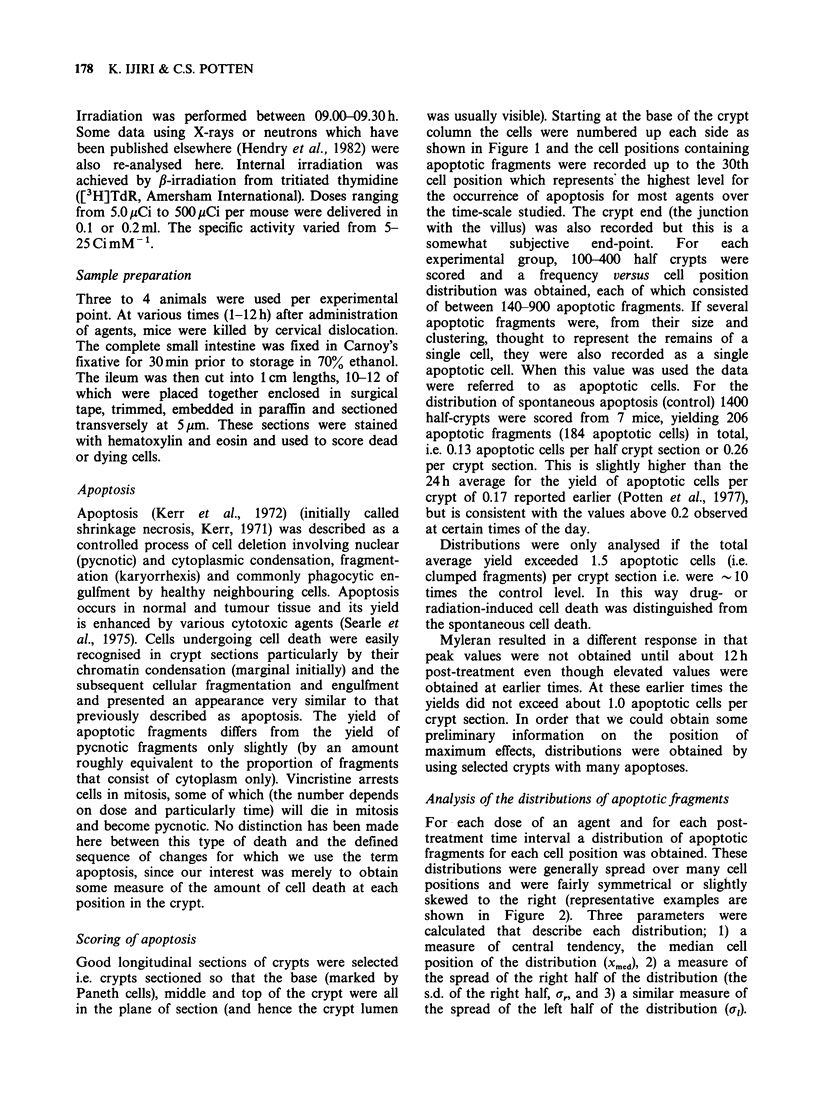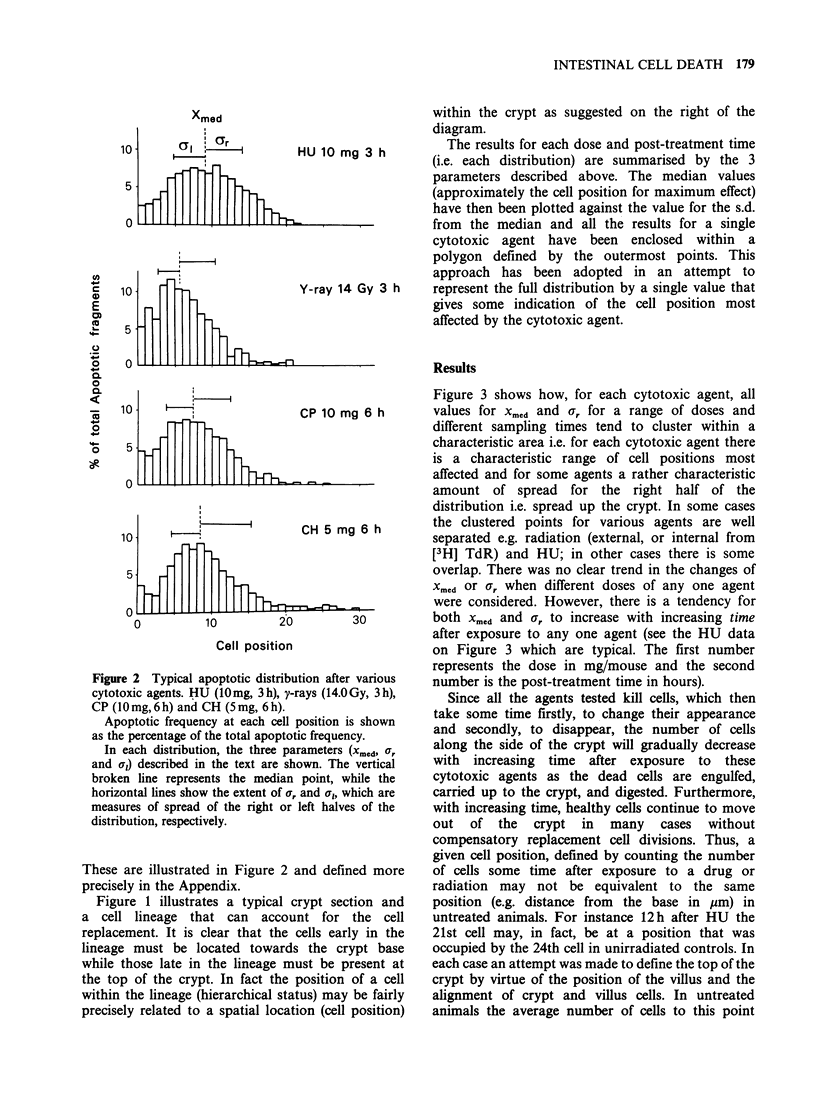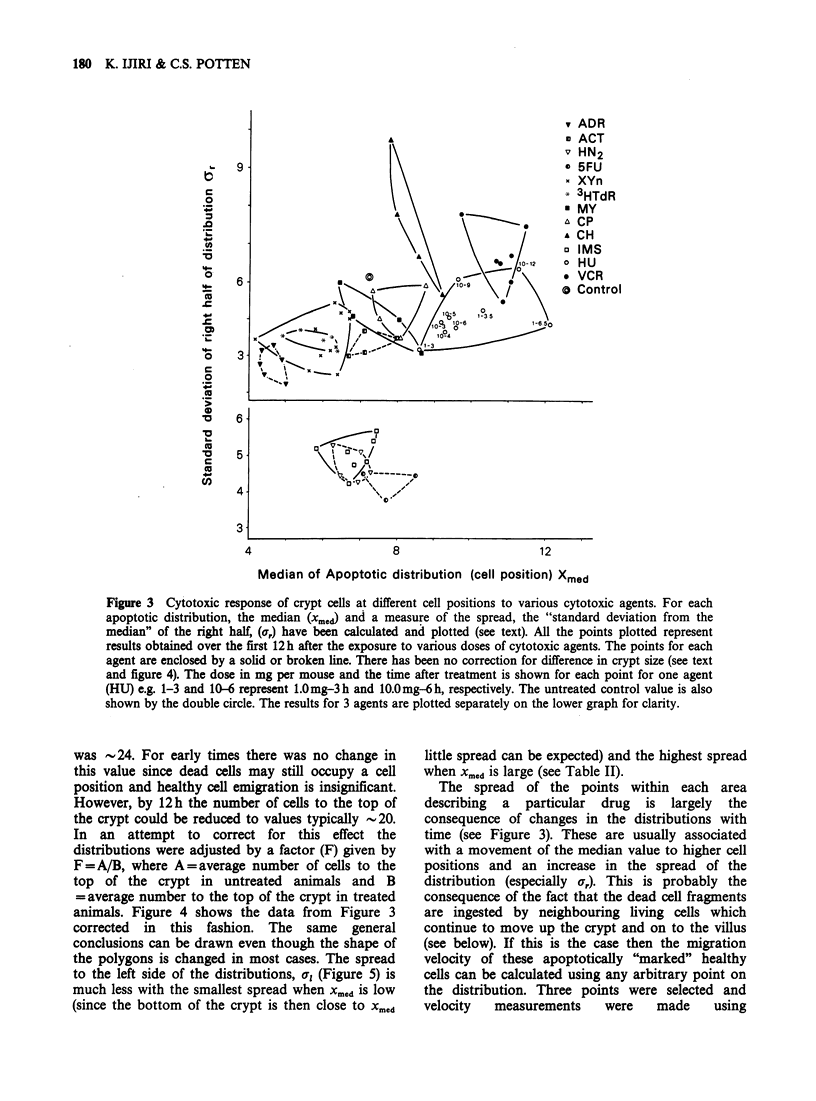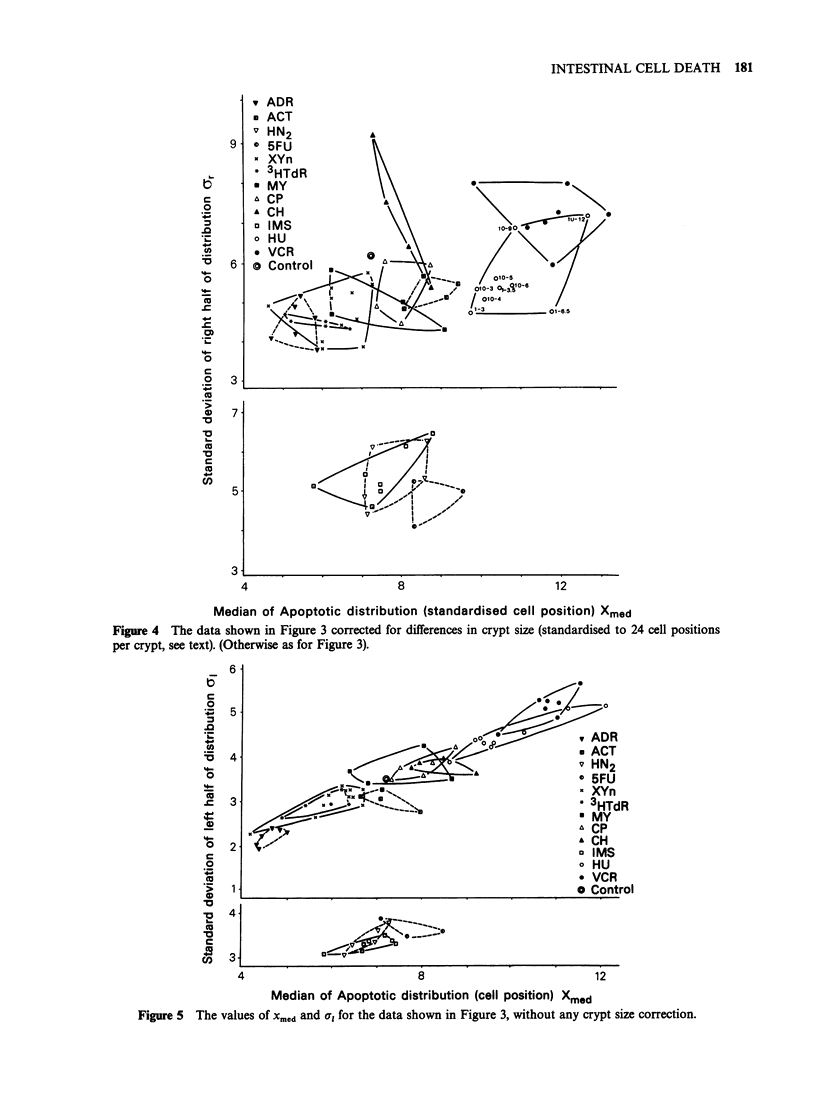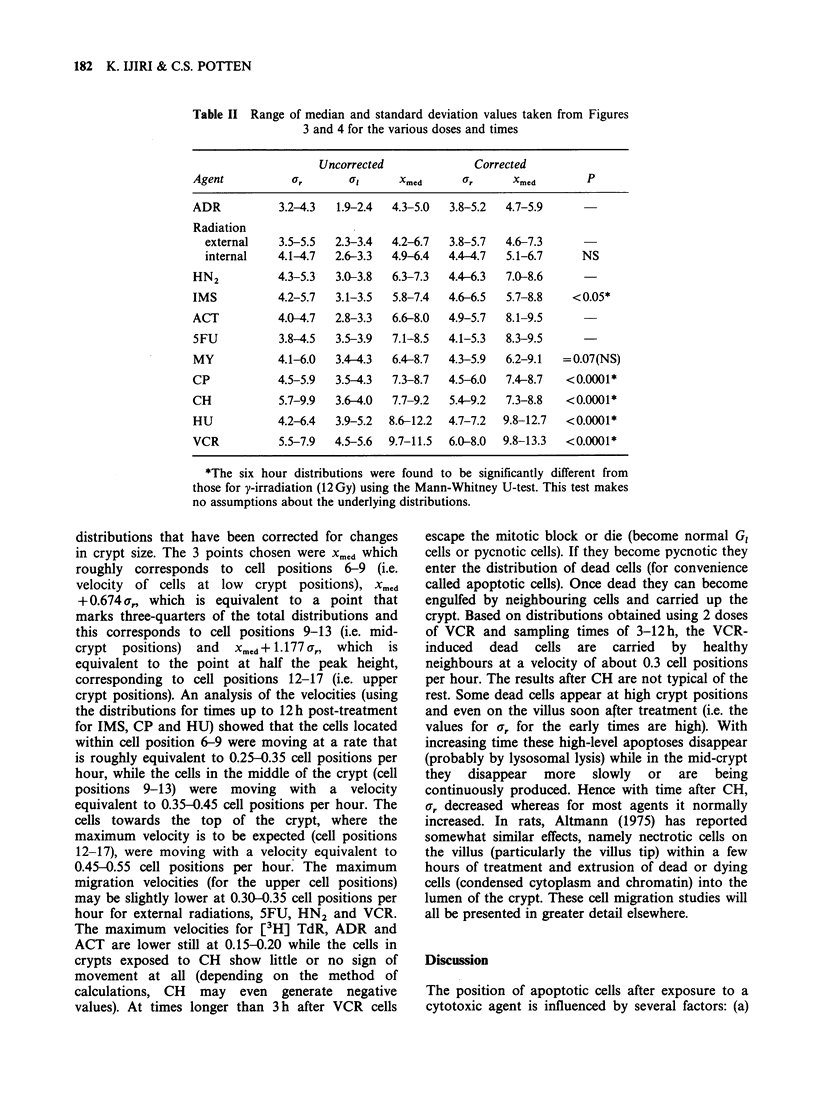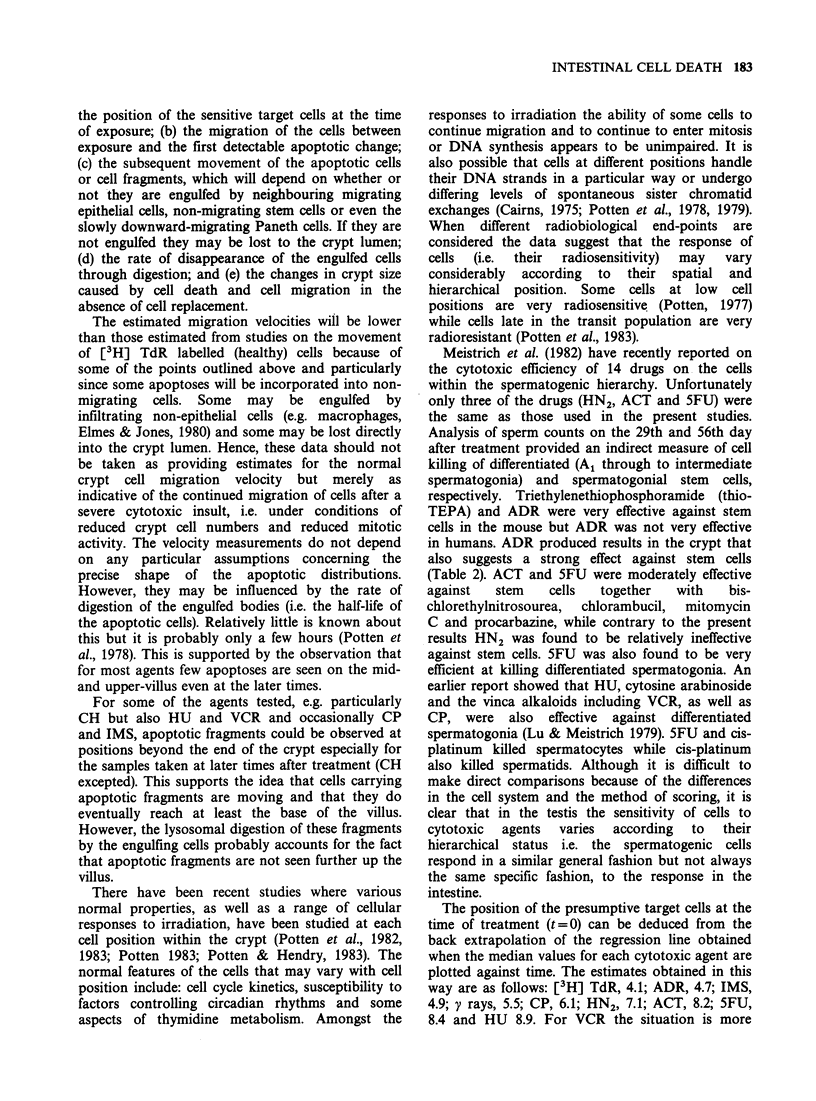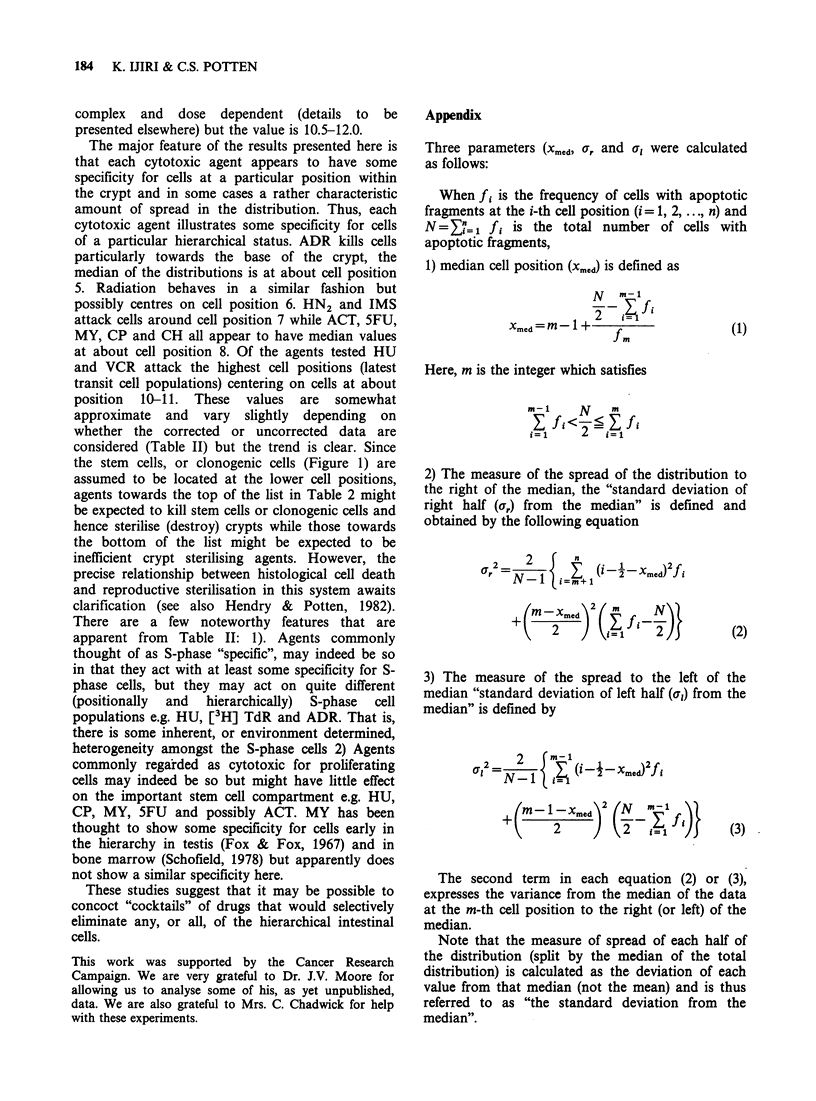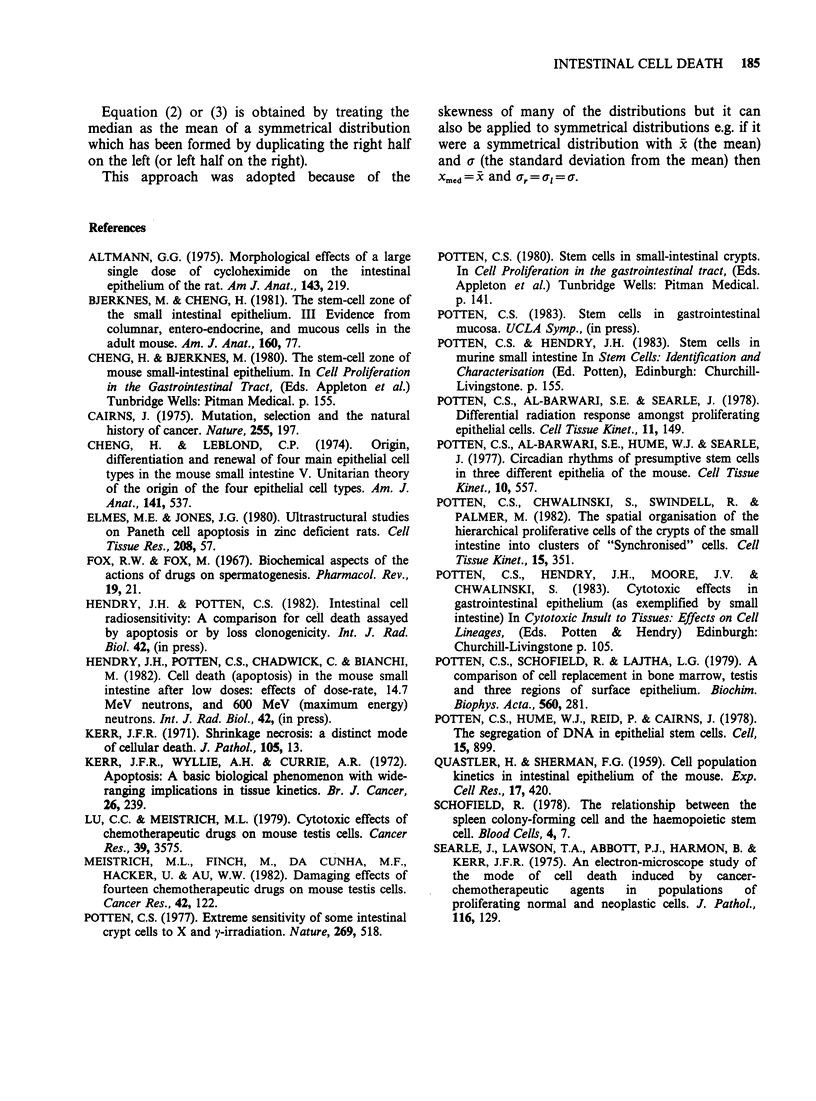# Response of intestinal cells of differing topographical and hierarchical status to ten cytotoxic drugs and five sources of radiation.

**DOI:** 10.1038/bjc.1983.25

**Published:** 1983-02

**Authors:** K. Ijiri, C. S. Potten

## Abstract

The spacial distribution of cell death among the epithelial cells lining the adult mammalian small intestinal mucosa at various times after a range of doses of 10 different drugs as well as after internal or external irradiation (beta particles from tritium, gamma- and X-rays and neutrons) has been recorded. Cell death, expressed as pycnosis or apoptosis, has been recorded for each cell position up the side of the crypts of the small intestine. The results, in the form of distributions of dead cells at each cell position, show that each of the various cytotoxic agents tends to act preferentially over a characteristic small range of cell positions. Since cell position is likely to be related to hierarchical cell position within a family tree or cell lineage, each agent tends to act with greatest efficiency on cells at a particular position within the lineage. Adriamycin and the various forms of radiation tend to kill cells preferentially at cell position 4-5 i.e. on cells very early in the lineage, probably stem cells. Isopropyl-methane-sulphonate, nitrogen mustard and possibly Actinomycin-D act on cell position 6-7, while 5-fluorouracil, Myleran, cyclophosphamide, and cycloheximide tend to kill cells at cell position 7-9. Vincristine and hydroxyurea are the 2 agents that exhibit a specificity for cells highest up the crypt, i.e. latest in transit population of the cell lineage by acting on cell positions 10 or 11. The data also suggest that normal healthy cells continue to migrate up the crypt and onto the villus in spite of considerable cell death and reduced cell production.


*Br. J. Cancer* (1983), **47**, 175–185

### Response of intestinal cells of differing topographical and hierarchical status to ten cytotoxic drugs and five sources of radiation

K. Ijiri* & C.S. Potten†

*Paterson Laboratories, Christie Hospital and Holt Radium Institute, Withington, Manchester M20 9BX.*

**Summary** The spacial distribution of cell death among the epithelial cells lining the adult mammalian small intestinal mucosa at various times after a range of doses of 10 different drugs as well as after internal or external irradiation ($\beta$ particles from tritium, $\gamma$- and X-rays and neutrons) has been recorded. Cell death, expressed as pycnosis or apoptosis, has been recorded for each cell position up the side of the crypts of the small intestine. The results, in the form of distributions of dead cells at each cell position, show that each of the various cytotoxic agents tends to act preferentially over a characteristic small range of cell positions. Since cell position is likely to be related to hierarchical cell position within a family tree or cell lineage, each agent tends to act with greatest efficiency on cells at a particular position within the lineage.

Adriamycin and the various forms of radiation tend to kill cells preferentially at cell position 4–5 i.e. on cells very early in the lineage, probably stem cells. Isopropyl-methane-sulphonate, nitrogen mustard and possibly Actinomycin-D act on cell position 6–7, while 5-fluorouracil, Myleran, cyclophosphamide, and cycloheximide tend to kill cells at cell position 7–9. Vincristine and hydroxyurea are the 2 agents that exhibit a specificity for cells highest up the crypt, i.e. latest in transit population of the cell lineage by acting on cell positions 10 or 11. The data also suggest that normal healthy cells continue to migrate up the crypt and onto the villus in spite of considerable cell death and reduced cell production.

Cytotoxic agents that are of therapeutic interest commonly act on proliferative cells or on proliferating cells at a particular phase of their cell cycle. For the most part it is assumed that all cells passing through the cell cycle will be equally affected. However, it is possible that cells of differing hierarchical status may respond in their own characteristic fashion with a characteristic sensitivity. This can be explored in the highly structured surface epithelia.

The epithelial cells lining the adult mammalian small intestinal mucosa (villi and crypts) represent an hierarchical cell lineage that can be related to the tissue architecture. The suggestion that cell replacement within the crypts of the small intestine can be described by a cell lineage, or series of cell lineages, with one or a relatively small number of lineage ancestor cells—stem cells—has been made by many authors (e.g. Quastler & Sherman, 1959; Cheng & Leblond, 1974; Potten 1980; Potten *et al.*, 1982) (see Figure 1). The precise number of non-migrating lineage ancestor cells remains uncertain but in mice is probably less than 16, the number of cells in a circumferential section through the crypt. From the general polarity of the tissue, cell migration, cell kinetic and regeneration studies, the position within the crypt of the various generations within the cell lineage can be deduced, with the stem cells being scattered amongst locations near the base of the crypt (i.e. positions below the fifth cell position) (Cheng & Leblond, 1974; Cheng & Bjerknes, 1980; Bjerknes & Cheng, 1981; Potten, 1980; Potten *et al.*, 1983; Potten & Hendry, 1983). The base of the crypt contains several mature and immature Paneth cells. The Paneth precursors and putative stem cells cycle at a slower rate ($T_c$ = 15–36 h) than the majority of proliferating crypt cells situated at higher positions ($T_c$ = 11 h for cell positions 6–20). Above these are several cell positions where no proliferative activity can be detected, i.e. the region of post-mitotic maturing columnar and goblet cells which migrate onto the villus where they perform their function, become senescent and are shed from the villus.

Thus, by studying the behaviour of cells at different positions within the crypt it is possible to study the behaviour of cells of different hierarchical status. Differences in hierarchical status (differentiation/maturation) might be expected to be associated with differences in cell cycle progression rate, microenvironment, microvasculature or cell metabolism and some, or all of these might result in differences in the response of cells to various cytotoxic agents. Here, dead or dying cells (recorded as the appearance of histologically recognisable

*Permanent address: Zoological Institute, Faculty of Science, University of Tokyo, Hongo, Tokyo 113, Japan.
†Correspondence: C.S. Potten, Paterson Laboratories, Christie Hospital and Holt Radium Institute, Manchester M20 9BX.
Received 16 July 1982; accepted 12 October 1982.

0007-0920/83/020175-11 $02.00
© The Macmillan Press Ltd., 1983

**Figure 1** Schematic representation of longitudinal section of a crypt showing the method of numbering the cells from the base to the top of the crypt. A = Apoptotic cells (or apoptotic fragments which are often clustered). P = Paneth cells with Paneth granules, G = Goblet cells, M = Mitosis, E = Enteroendocrine cell, S = Stem cells, F = Pericryptal Fibroblast. The proliferative zone is indicated as well at the stem cell region. On the right a possible cell lineage for cell replacement in the crypt is shown. The entire crypt could be regarded as originating from a single primary (ultimate) stem cell (S′) in which case there would be 7 amplification divisions (generations). Alternatively there may be about 12 lineage-originators (stem cells) e.g. those cells within the lower dashed box. In this case 3 amplifying transit divisions would be expected. All the cells within the lower dashed box could be regarded as identical stem cells (S). However, they may be positioned at different levels (a–e) in relationship to a hypothetical stem cell milieu or focal point (a). The position relative to the focal point may determine the cell cycle duration ($G_0$ duration) and self-maintenance (differentiation) probabilities (see Potten *et al.*, 1979). This operational heterogeneity may extend even further to include more cells (lower and upper dashed boxes) e.g. the clonogenic fraction (~ 80 cells). In this case only one further amplifying cell division would be expected. The diagram reflects the uncertainty in our knowledge on the precise number of stem cells. The approximate cell position within the crypt for each cell generation is also indicated (right hand side).

dead cells, apoptosis, within a short time of treatment) have been scored at each cell position within crypts at various times after exposure to a wide range of cytotoxic agents. The results show that each agent has a certain selectivity for a particular cell position along the crypt and hence hierarchical status.

### Materials and methods

### *Animals*

Ten–12-week-old male B6D2$F_1$ (Pat) mice (~25 g) were used throughout the experiments. The animals were kept under a 12 h dark (18.00–06.00), 12 h light regimen, and were given food and water *ad libitum.*

### *Drugs*

All drug solutions were made up immediately before use, and dilutions were carried out either in 0.9% saline or in sterile water (except for Myleran, which was dissolved in arachis oil). All injections involved 0.2 ml given i.p. at 09.00 h. Drug doses injected in this volume are quoted as mg per mouse (1 mg per mouse is ~40 mg $kg^{-1}$). Cytotoxic drugs used were: isopropyl–methane–sulphonate (IMS, Koch-Light, Colnbrook); Myleran (R), (MY, Burroughs-Wellcome, busulphan, 1, 4-dimethane sulphonyl oxybutane); cyclophosphamide (CP, Endoxana (R), WB Pharmaceuticals, Bracknell), vincristine (VCR, Oncovin (R), Lilly, Basingstoke); hydroxyurea (HU, Sigma, Poole) and cycloheximide (CH, Sigma, Poole). The results presented here for mechlorethamine ($HN_2$, mustine hydrochloride, Boots, Nottingham); 5-fluorouracil (5FU, Fluorouracil (R), Roche, Welwyn Garden City); actinomycin-D (ACT, Cosmogen (R), Merck Sharp and Dohme) and adriamycin (ADR, Montedison, Barnet, Hertfordshire) are unpublished results from

**Table I** Cytotoxic agents, range of doses and post-treatment sampling times for which apoptotic distributions were obtained

| *Agent* | *Dose per mouse* | *Time of sampling after treatment (h)* |
|---|---|---|
| *Alkylating agents* | | |
| IMS | 0.1 mg | $3^+$ |
| | 1.0 | 3, 6, 12 |
| | 10.0 | 3, 6, 9, 12 |
| MY | 1.0 mg | 6, 9, 12 |
| | 3.0 | 5.5 |
| CP | 0.1 mg | $3^+$ |
| | 1.0 | 9, 12 |
| | 10.0 | $3^+$,6,9,12 |
| $HN_2$ | 0.043 mg* | $1^+$,3,5,7,10,12 |
| *Antimetabolites* | | |
| 5FU | 2.9 mg* | $1^+$,$3^+$,$5^+$,7,10,12 |
| HU | 0.1 mg | $3^+$,$6^+$ |
| | 1.0 | 3, 3.5, 6.5 |
| | 10.0 | 3, 4, 5, 6, 9, 12 |
| CH | 5.0 | $1^+$,2,3,6,12 |
| *Antibiotics* | | |
| ACT | 0.017 mg* | $1^+$,$3^+$,5,7,9,12 |
| ADR | 0.23 mg* | $1^+$,3,5,7,9,11,12 |
| *Strathmokinetic agents* | | |
| VCR | 0.01 mg | $3^+$,6,9,12 |
| | 0.1 | 3, 6, 9, 12 |
| *Radiation* | | |
| Internal $\beta$[$^3$H] TdR | 5.0 $\mu$Ci | $6^+$ |
| | 50 | $3.5^+$,6,6.5,9,12 |
| | 500 | 6 |
| External $^{137}$Cs $\gamma$-rays | 0.22 Gy (4.5 Gy min$^{-1}$) | 3 |
| | 0.63 | 3 |
| | 12.0 | 3.6, 9, 12 |
| | 14.0 | 3, 6 |
| 300 kV$_p$ X-rays | 0.005 Gy (0.6 Gy min$^{-1}$)$^a$ | $3^+$ |
| | 0.09 | $3^+$ |
| | 0.15 | 3 |
| 14.7 MeV neutrons | 0.075 Gy (0.005 Gy min$^{-1}$)$^a$ | 3 |
| 600 MeV neutrons | 0.15 (0.0027 Gy min$^{-1}$)$^a$ | 3 |

*Data kindly supplied by Dr. J.V. Moore.
[a]Full description of the technical details are presented elsewhere (Hendry *et al.*, 1982).
$^+$Distributions not analysed because the absolute yield was too low (i.e. <1.5 apoptotic cells per crypt section).

Dr. J.V. Moore which will be presented in full elsewhere. The data were recorded in the same way but slightly different criteria were used to define dead or dying cells. Table I shows the range of doses and post-treatment sampling times studied.

### *Irradiation*

External irradiation was achieved using a $^{137}$Cs-$\gamma$-irradiator designed for whole-body irradiation of small animals at a dose rate of 4.5 Gy min$^{-1}$.

Irradiation was performed between 09.00–09.30 h. Some data using X-rays or neutrons which have been published elsewhere (Hendry *et al.*, 1982) were also re-analysed here. Internal irradiation was achieved by $\beta$-irradiation from tritiated thymidine ([$^3$H]TdR, Amersham International). Doses ranging from 5.0 $\mu$Ci to 500 $\mu$Ci per mouse were delivered in 0.1 or 0.2 ml. The specific activity varied from 5–25 Ci mM$^{-1}$.

*Sample preparation*

Three to 4 animals were used per experimental point. At various times (1–12 h) after administration of agents, mice were killed by cervical dislocation. The complete small intestine was fixed in Carnoy's fixative for 30 min prior to storage in 70% ethanol. The ileum was then cut into 1 cm lengths, 10–12 of which were placed together enclosed in surgical tape, trimmed, embedded in paraffin and sectioned transversely at 5 $\mu$m. These sections were stained with hematoxylin and eosin and used to score dead or dying cells.

*Apoptosis*

Apoptosis (Kerr *et al.*, 1972) (initially called shrinkage necrosis, Kerr, 1971) was described as a controlled process of cell deletion involving nuclear (pycnotic) and cytoplasmic condensation, fragmentation (karyorrhexis) and commonly phagocytic engulfment by healthy neighbouring cells. Apoptosis occurs in normal and tumour tissue and its yield is enhanced by various cytotoxic agents (Searle *et al.*, 1975). Cells undergoing cell death were easily recognised in crypt sections particularly by their chromatin condensation (marginal initially) and the subsequent cellular fragmentation and engulfment and presented an appearance very similar to that previously described as apoptosis. The yield of apoptotic fragments differs from the yield of pycnotic fragments only slightly (by an amount roughly equivalent to the proportion of fragments that consist of cytoplasm only). Vincristine arrests cells in mitosis, some of which (the number depends on dose and particularly time) will die in mitosis and become pycnotic. No distinction has been made here between this type of death and the defined sequence of changes for which we use the term apoptosis, since our interest was merely to obtain some measure of the amount of cell death at each position in the crypt.

*Scoring of apoptosis*

Good longitudinal sections of crypts were selected i.e. crypts sectioned so that the base (marked by Paneth cells), middle and top of the crypt were all in the plane of section (and hence the crypt lumen was usually visible). Starting at the base of the crypt column the cells were numbered up each side as shown in Figure 1 and the cell positions containing apoptotic fragments were recorded up to the 30th cell position which represents the highest level for the occurrence of apoptosis for most agents over the time-scale studied. The crypt end (the junction with the villus) was also recorded but this is a somewhat subjective end-point. For each experimental group, 100–400 half crypts were scored and a frequency *versus* cell position distribution was obtained, each of which consisted of between 140–900 apoptotic fragments. If several apoptotic fragments were, from their size and clustering, thought to represent the remains of a single cell, they were also recorded as a single apoptotic cell. When this value was used the data were referred to as apoptotic cells. For the distribution of spontaneous apoptosis (control) 1400 half-crypts were scored from 7 mice, yielding 206 apoptotic fragments (184 apoptotic cells) in total, i.e. 0.13 apoptotic cells per half crypt section or 0.26 per crypt section. This is slightly higher than the 24 h average for the yield of apoptotic cells per crypt of 0.17 reported earlier (Potten *et al.*, 1977), but is consistent with the values above 0.2 observed at certain times of the day.

Distributions were only analysed if the total average yield exceeded 1.5 apoptotic cells (i.e. clumped fragments) per crypt section i.e. were $\sim$10 times the control level. In this way drug- or radiation-induced cell death was distinguished from the spontaneous cell death.

Myleran resulted in a different response in that peak values were not obtained until about 12 h post-treatment even though elevated values were obtained at earlier times. At these earlier times the yields did not exceed about 1.0 apoptotic cells per crypt section. In order that we could obtain some preliminary information on the position of maximum effects, distributions were obtained by using selected crypts with many apoptoses.

*Analysis of the distributions of apoptotic fragments*

For each dose of an agent and for each post-treatment time interval a distribution of apoptotic fragments for each cell position was obtained. These distributions were generally spread over many cell positions and were fairly symmetrical or slightly skewed to the right (representative examples are shown in Figure 2). Three parameters were calculated that describe each distribution; 1) a measure of central tendency, the median cell position of the distribution ($x_{med}$), 2) a measure of the spread of the right half of the distribution (the s.d. of the right half, $\sigma_r$, and 3) a similar measure of the spread of the left half of the distribution ($\sigma_l$).

**Figure 2** Typical apoptotic distribution after various cytotoxic agents. HU (10 mg, 3 h), γ-rays (14.0 Gy, 3 h), CP (10 mg, 6 h) and CH (5 mg, 6 h).

Apoptotic frequency at each cell position is shown as the percentage of the total apoptotic frequency.

In each distribution, the three parameters ($x_{med}$, $\sigma_r$ and $\sigma_l$) described in the text are shown. The vertical broken line represents the median point, while the horizontal lines show the extent of $\sigma_r$ and $\sigma_l$, which are measures of spread of the right or left halves of the distribution, respectively.

These are illustrated in Figure 2 and defined more precisely in the Appendix.

Figure 1 illustrates a typical crypt section and a cell lineage that can account for the cell replacement. It is clear that the cells early in the lineage must be located towards the crypt base while those late in the lineage must be present at the top of the crypt. In fact the position of a cell within the lineage (hierarchical status) may be fairly precisely related to a spatial location (cell position) within the crypt as suggested on the right of the diagram.

The results for each dose and post-treatment time (i.e. each distribution) are summarised by the 3 parameters described above. The median values (approximately the cell position for maximum effect) have then been plotted against the value for the s.d. from the median and all the results for a single cytotoxic agent have been enclosed within a polygon defined by the outermost points. This approach has been adopted in an attempt to represent the full distribution by a single value that gives some indication of the cell position most affected by the cytotoxic agent.

### Results

Figure 3 shows how, for each cytotoxic agent, all values for $x_{med}$ and $\sigma_r$ for a range of doses and different sampling times tend to cluster within a characteristic area i.e. for each cytotoxic agent there is a characteristic range of cell positions most affected and for some agents a rather characteristic amount of spread for the right half of the distribution i.e. spread up the crypt. In some cases the clustered points for various agents are well separated e.g. radiation (external, or internal from [$^3$H] TdR) and HU; in other cases there is some overlap. There was no clear trend in the changes of $x_{med}$ or $\sigma_r$ when different doses of any one agent were considered. However, there is a tendency for both $x_{med}$ and $\sigma_r$ to increase with increasing *time* after exposure to any one agent (see the HU data on Figure 3 which are typical. The first number represents the dose in mg/mouse and the second number is the post-treatment time in hours).

Since all the agents tested kill cells, which then take some time firstly, to change their appearance and secondly, to disappear, the number of cells along the side of the crypt will gradually decrease with increasing time after exposure to these cytotoxic agents as the dead cells are engulfed, carried up to the crypt, and digested. Furthermore, with increasing time, healthy cells continue to move out of the crypt in many cases without compensatory replacement cell divisions. Thus, a given cell position, defined by counting the number of cells some time after exposure to a drug or radiation may not be equivalent to the same position (e.g. distance from the base in μm) in untreated animals. For instance 12 h after HU the 21st cell may, in fact, be at a position that was occupied by the 24th cell in unirradiated controls. In each case an attempt was made to define the top of the crypt by virtue of the position of the villus and the alignment of crypt and villus cells. In untreated animals the average number of cells to this point

**Figure 3** Cytotoxic response of crypt cells at different cell positions to various cytotoxic agents. For each apoptotic distribution, the median ($x_{med}$) and a measure of the spread, the "standard deviation from the median" of the right half, ($\sigma_r$) have been calculated and plotted (see text). All the points plotted represent results obtained over the first 12 h after the exposure to various doses of cytotoxic agents. The points for each agent are enclosed by a solid or broken line. There has been no correction for difference in crypt size (see text and figure 4). The dose in mg per mouse and the time after treatment is shown for each point for one agent (HU) e.g. 1–3 and 10–6 represent 1.0 mg–3 h and 10.0 mg–6 h, respectively. The untreated control value is also shown by the double circle. The results for 3 agents are plotted separately on the lower graph for clarity.

was ~24. For early times there was no change in this value since dead cells may still occupy a cell position and healthy cell emigration is insignificant. However, by 12 h the number of cells to the top of the crypt could be reduced to values typically ~20. In an attempt to correct for this effect the distributions were adjusted by a factor (F) given by F = A/B, where A = average number of cells to the top of the crypt in untreated animals and B = average number to the top of the crypt in treated animals. Figure 4 shows the data from Figure 3 corrected in this fashion. The same general conclusions can be drawn even though the shape of the polygons is changed in most cases. The spread to the left side of the distributions, $\sigma_l$ (Figure 5) is much less with the smallest spread when $x_{med}$ is low (since the bottom of the crypt is then close to $x_{med}$ little spread can be expected) and the highest spread when $x_{med}$ is large (see Table II).

The spread of the points within each area describing a particular drug is largely the consequence of changes in the distributions with time (see Figure 3). These are usually associated with a movement of the median value to higher cell positions and an increase in the spread of the distribution (especially $\sigma_r$). This is probably the consequence of the fact that the dead cell fragments are ingested by neighbouring living cells which continue to move up the crypt and on to the villus (see below). If this is the case then the migration velocity of these apoptotically "marked" healthy cells can be calculated using any arbitrary point on the distribution. Three points were selected and velocity measurements were made using

**Figure 4** The data shown in Figure 3 corrected for differences in crypt size (standardised to 24 cell positions per crypt, see text). (Otherwise as for Figure 3).

**Figure 5** The values of $x_{med}$ and $\sigma_l$ for the data shown in Figure 3, without any crypt size correction.

**Table II** Range of median and standard deviation values taken from Figures 3 and 4 for the various doses and times

| Agent | Uncorrected $\sigma_r$ | Uncorrected $\sigma_l$ | Uncorrected $x_{med}$ | Corrected $\sigma_r$ | Corrected $x_{med}$ | P |
|---|---|---|---|---|---|---|
| ADR | 3.2–4.3 | 1.9–2.4 | 4.3–5.0 | 3.8–5.2 | 4.7–5.9 | — |
| Radiation | | | | | | |
| external | 3.5–5.5 | 2.3–3.4 | 4.2–6.7 | 3.8–5.7 | 4.6–7.3 | — |
| internal | 4.1–4.7 | 2.6–3.3 | 4.9–6.4 | 4.4–4.7 | 5.1–6.7 | NS |
| $HN_2$ | 4.3–5.3 | 3.0–3.8 | 6.3–7.3 | 4.4–6.3 | 7.0–8.6 | — |
| IMS | 4.2–5.7 | 3.1–3.5 | 5.8–7.4 | 4.6–6.5 | 5.7–8.8 | <0.05* |
| ACT | 4.0–4.7 | 2.8–3.3 | 6.6–8.0 | 4.9–5.7 | 8.1–9.5 | — |
| 5FU | 3.8–4.5 | 3.5–3.9 | 7.1–8.5 | 4.1–5.3 | 8.3–9.5 | — |
| MY | 4.1–6.0 | 3.4–4.3 | 6.4–8.7 | 4.3–5.9 | 6.2–9.1 | =0.07(NS) |
| CP | 4.5–5.9 | 3.5–4.3 | 7.3–8.7 | 4.5–6.0 | 7.4–8.7 | <0.0001* |
| CH | 5.7–9.9 | 3.6–4.0 | 7.7–9.2 | 5.4–9.2 | 7.3–8.8 | <0.0001* |
| HU | 4.2–6.4 | 3.9–5.2 | 8.6–12.2 | 4.7–7.2 | 9.8–12.7 | <0.0001* |
| VCR | 5.5–7.9 | 4.5–5.6 | 9.7–11.5 | 6.0–8.0 | 9.8–13.3 | <0.0001* |

*The six hour distributions were found to be significantly different from those for γ-irradiation (12 Gy) using the Mann-Whitney U-test. This test makes no assumptions about the underlying distributions.

distributions that have been corrected for changes in crypt size. The 3 points chosen were $x_{med}$ which roughly corresponds to cell positions 6–9 (i.e. velocity of cells at low crypt positions), $x_{med}+0.674\sigma_r$, which is equivalent to a point that marks three-quarters of the total distributions and this corresponds to cell positions 9–13 (i.e. mid-crypt positions) and $x_{med}+1.177\sigma_r$, which is equivalent to the point at half the peak height, corresponding to cell positions 12–17 (i.e. upper crypt positions). An analysis of the velocities (using the distributions for times up to 12 h post-treatment for IMS, CP and HU) showed that the cells located within cell position 6–9 were moving at a rate that is roughly equivalent to 0.25–0.35 cell positions per hour, while the cells in the middle of the crypt (cell positions 9–13) were moving with a velocity equivalent to 0.35–0.45 cell positions per hour. The cells towards the top of the crypt, where the maximum velocity is to be expected (cell positions 12–17), were moving with a velocity equivalent to 0.45–0.55 cell positions per hour. The maximum migration velocities (for the upper cell positions) may be slightly lower at 0.30–0.35 cell positions per hour for external radiations, 5FU, $HN_2$ and VCR. The maximum velocities for [$^3$H] TdR, ADR and ACT are lower still at 0.15–0.20 while the cells in crypts exposed to CH show little or no sign of movement at all (depending on the method of calculations, CH may even generate negative values). At times longer than 3 h after VCR cells escape the mitotic block or die (become normal $G_l$ cells or pycnotic cells). If they become pycnotic they enter the distribution of dead cells (for convenience called apoptotic cells). Once dead they can become engulfed by neighbouring cells and carried up the crypt. Based on distributions obtained using 2 doses of VCR and sampling times of 3–12 h, the VCR-induced dead cells are carried by healthy neighbours at a velocity of about 0.3 cell positions per hour. The results after CH are not typical of the rest. Some dead cells appear at high crypt positions and even on the villus soon after treatment (i.e. the values for $\sigma_r$ for the early times are high). With increasing time these high-level apoptoses disappear (probably by lysosomal lysis) while in the mid-crypt they disappear more slowly or are being continuously produced. Hence with time after CH, $\sigma_r$ decreased whereas for most agents it normally increased. In rats, Altmann (1975) has reported somewhat similar effects, namely nectrotic cells on the villus (particularly the villus tip) within a few hours of treatment and extrusion of dead or dying cells (condensed cytoplasm and chromatin) into the lumen of the crypt. These cell migration studies will all be presented in greater detail elsewhere.

### Discussion

The position of apoptotic cells after exposure to a cytotoxic agent is influenced by several factors: (a)

the position of the sensitive target cells at the time of exposure; (b) the migration of the cells between exposure and the first detectable apoptotic change; (c) the subsequent movement of the apoptotic cells or cell fragments, which will depend on whether or not they are engulfed by neighbouring migrating epithelial cells, non-migrating stem cells or even the slowly downward-migrating Paneth cells. If they are not engulfed they may be lost to the crypt lumen; (d) the rate of disappearance of the engulfed cells through digestion; and (e) the changes in crypt size caused by cell death and cell migration in the absence of cell replacement.

The estimated migration velocities will be lower than those estimated from studies on the movement of [$^3$H] TdR labelled (healthy) cells because of some of the points outlined above and particularly since some apoptoses will be incorporated into non-migrating cells. Some may be engulfed by infiltrating non-epithelial cells (e.g. macrophages, Elmes & Jones, 1980) and some may be lost directly into the crypt lumen. Hence, these data should not be taken as providing estimates for the normal crypt cell migration velocity but merely as indicative of the continued migration of cells after a severe cytotoxic insult, i.e. under conditions of reduced crypt cell numbers and reduced mitotic activity. The velocity measurements do not depend on any particular assumptions concerning the precise shape of the apoptotic distributions. However, they may be influenced by the rate of digestion of the engulfed bodies (i.e. the half-life of the apoptotic cells). Relatively little is known about this but it is probably only a few hours (Potten *et al.*, 1978). This is supported by the observation that for most agents few apoptoses are seen on the mid- and upper-villus even at the later times.

For some of the agents tested, e.g. particularly CH but also HU and VCR and occasionally CP and IMS, apoptotic fragments could be observed at positions beyond the end of the crypt especially for the samples taken at later times after treatment (CH excepted). This supports the idea that cells carrying apoptotic fragments are moving and that they do eventually reach at least the base of the villus. However, the lysosomal digestion of these fragments by the engulfing cells probably accounts for the fact that apoptotic fragments are not seen further up the villus.

There have been recent studies where various normal properties, as well as a range of cellular responses to irradiation, have been studied at each cell position within the crypt (Potten *et al.*, 1982, 1983; Potten 1983; Potten & Hendry, 1983). The normal features of the cells that may vary with cell position include: cell cycle kinetics, susceptibility to factors controlling circadian rhythms and some aspects of thymidine metabolism. Amongst the responses to irradiation the ability of some cells to continue migration and to continue to enter mitosis or DNA synthesis appears to be unimpaired. It is also possible that cells at different positions handle their DNA strands in a particular way or undergo differing levels of spontaneous sister chromatid exchanges (Cairns, 1975; Potten *et al.*, 1978, 1979). When different radiobiological end-points are considered the data suggest that the response of cells (i.e. their radiosensitivity) may vary considerably according to their spatial and hierarchical position. Some cells at low cell positions are very radiosensitive (Potten, 1977) while cells late in the transit population are very radioresistant (Potten *et al.*, 1983).

Meistrich *et al.* (1982) have recently reported on the cytotoxic efficiency of 14 drugs on the cells within the spermatogenic hierarchy. Unfortunately only three of the drugs ($HN_2$, ACT and 5FU) were the same as those used in the present studies. Analysis of sperm counts on the 29th and 56th day after treatment provided an indirect measure of cell killing of differentiated ($A_1$ through to intermediate spermatogonia) and spermatogonial stem cells, respectively. Triethylenethiophosphoramide (thio-TEPA) and ADR were very effective against stem cells in the mouse but ADR was not very effective in humans. ADR produced results in the crypt that also suggests a strong effect against stem cells (Table 2). ACT and 5FU were moderately effective against stem cells together with bis-chlorethylnitrosourea, chlorambucil, mitomycin C and procarbazine, while contrary to the present results $HN_2$ was found to be relatively ineffective against stem cells. 5FU was also found to be very efficient at killing differentiated spermatogonia. An earlier report showed that HU, cytosine arabinoside and the vinca alkaloids including VCR, as well as CP, were also effective against differentiated spermatogonia (Lu & Meistrich 1979). 5FU and cis-platinum killed spermatocytes while cis-platinum also killed spermatids. Although it is difficult to make direct comparisons because of the differences in the cell system and the method of scoring, it is clear that in the testis the sensitivity of cells to cytotoxic agents varies according to their hierarchical status i.e. the spermatogenic cells respond in a similar general fashion but not always the same specific fashion, to the response in the intestine.

The position of the presumptive target cells at the time of treatment ($t = 0$) can be deduced from the back extrapolation of the regression line obtained when the median values for each cytotoxic agent are plotted against time. The estimates obtained in this way are as follows: [$^3$H] TdR, 4.1; ADR, 4.7; IMS, 4.9; $\gamma$ rays, 5.5; CP, 6.1; $HN_2$, 7.1; ACT, 8.2; 5FU, 8.4 and HU 8.9. For VCR the situation is more complex and dose dependent (details to be presented elsewhere) but the value is 10.5–12.0.

The major feature of the results presented here is that each cytotoxic agent appears to have some specificity for cells at a particular position within the crypt and in some cases a rather characteristic amount of spread in the distribution. Thus, each cytotoxic agent illustrates some specificity for cells of a particular hierarchical status. ADR kills cells particularly towards the base of the crypt, the median of the distributions is at about cell position 5. Radiation behaves in a similar fashion but possibly centres on cell position 6. $HN_2$ and IMS attack cells around cell position 7 while ACT, 5FU, MY, CP and CH all appear to have median values at about cell position 8. Of the agents tested HU and VCR attack the highest cell positions (latest transit cell populations) centering on cells at about position 10–11. These values are somewhat approximate and vary slightly depending on whether the corrected or uncorrected data are considered (Table II) but the trend is clear. Since the stem cells, or clonogenic cells (Figure 1) are assumed to be located at the lower cell positions, agents towards the top of the list in Table 2 might be expected to kill stem cells or clonogenic cells and hence sterilise (destroy) crypts while those towards the bottom of the list might be expected to be inefficient crypt sterilising agents. However, the precise relationship between histological cell death and reproductive sterilisation in this system awaits clarification (see also Hendry & Potten, 1982). There are a few noteworthy features that are apparent from Table II: 1). Agents commonly thought of as S-phase "specific", may indeed be so in that they act with at least some specificity for S-phase cells, but they may act on quite different (positionally and hierarchically) S-phase cell populations e.g. HU, [$^3$H] TdR and ADR. That is, there is some inherent, or environment determined, heterogeneity amongst the S-phase cells 2) Agents commonly regarded as cytotoxic for proliferating cells may indeed be so but might have little effect on the important stem cell compartment e.g. HU, CP, MY, 5FU and possibly ACT. MY has been thought to show some specificity for cells early in the hierarchy in testis (Fox & Fox, 1967) and in bone marrow (Schofield, 1978) but apparently does not show a similar specificity here.

These studies suggest that it may be possible to concoct "cocktails" of drugs that would selectively eliminate any, or all, of the hierarchical intestinal cells.

This work was supported by the Cancer Research Campaign. We are very grateful to Dr. J.V. Moore for allowing us to analyse some of his, as yet unpublished, data. We are also grateful to Mrs. C. Chadwick for help with these experiments.

### Appendix

Three parameters ($x_{med}$, $\sigma_r$ and $\sigma_l$ were calculated as follows:

When $f_i$ is the frequency of cells with apoptotic fragments at the $i$-th cell position ($i = 1, 2, \ldots, n$) and $N = \sum_{i=1}^{n} f_i$ is the total number of cells with apoptotic fragments,

1) median cell position ($x_{med}$) is defined as

$$x_{med} = m - 1 + \frac{\frac{N}{2} - \sum_{i=1}^{m-1} f_i}{f_m} \quad (1)$$

Here, $m$ is the integer which satisfies

$$\sum_{i=1}^{m-1} f_i < \frac{N}{2} \leq \sum_{i=1}^{m} f_i$$

2) The measure of the spread of the distribution to the right of the median, the "standard deviation of right half ($\sigma_r$) from the median" is defined and obtained by the following equation

$$\sigma_r^2 = \frac{2}{N-1} \left\{ \sum_{i=m+1}^{n} (i - \tfrac{1}{2} - x_{med})^2 f_i + \left(\frac{m - x_{med}}{2}\right)^2 \left(\sum_{i=1}^{m} f_i - \frac{N}{2}\right) \right\} \quad (2)$$

3) The measure of the spread to the left of the median "standard deviation of left half ($\sigma_l$) from the median" is defined by

$$\sigma_l^2 = \frac{2}{N-1} \left\{ \sum_{i=1}^{m-1} (i - \tfrac{1}{2} - x_{med})^2 f_i + \left(\frac{m - 1 - x_{med}}{2}\right)^2 \left(\frac{N}{2} - \sum_{i=1}^{m-1} f_i\right) \right\} \quad (3)$$

The second term in each equation (2) or (3), expresses the variance from the median of the data at the $m$-th cell position to the right (or left) of the median.

Note that the measure of spread of each half of the distribution (split by the median of the total distribution) is calculated as the deviation of each value from that median (not the mean) and is thus referred to as "the standard deviation from the median".

Equation (2) or (3) is obtained by treating the median as the mean of a symmetrical distribution which has been formed by duplicating the right half on the left (or left half on the right).

This approach was adopted because of the skewness of many of the distributions but it can also be applied to symmetrical distributions e.g. if it were a symmetrical distribution with $\bar{x}$ (the mean) and $\sigma$ (the standard deviation from the mean) then $x_{med} = \bar{x}$ and $\sigma_r = \sigma_l = \sigma$.

**References**

ALTMANN, G.G. (1975). Morphological effects of a large single dose of cycloheximide on the intestinal epithelium of the rat. *Am J. Anat.*, **143**, 219.

BJERKNES, M. & CHENG, H. (1981). The stem-cell zone of the small intestinal epithelium. III Evidence from columnar, entero-endocrine, and mucous cells in the adult mouse. *Am. J. Anat.*, **160**, 77.

CHENG, H. & BJERKNES, M. (1980). The stem-cell zone of mouse small-intestinal epithelium. In *Cell Proliferation in the Gastrointestinal Tract,* (Eds. Appleton *et al.*) Tunbridge Wells: Pitman Medical. p. 155.

CAIRNS, J. (1975). Mutation, selection and the natural history of cancer. *Nature,* **255,** 197.

CHENG, H. & LEBLOND, C.P. (1974). Origin, differentiation and renewal of four main epithelial cell types in the mouse small intestine V. Unitarian theory of the origin of the four epithelial cell types. *Am. J. Anat.*, **141**, 537.

ELMES, M.E. & JONES, J.G. (1980). Ultrastructural studies on Paneth cell apoptosis in zinc deficient rats. *Cell Tissue Res.*, **208**, 57.

FOX, R.W. & FOX, M. (1967). Biochemical aspects of the actions of drugs on spermatogenesis. *Pharmacol. Rev.*, **19**, 21.

HENDRY, J.H. & POTTEN, C.S. (1982). Intestinal cell radiosensitivity: A comparison for cell death assayed by apoptosis or by loss clonogenicity. *Int. J. Rad. Biol.* **42**, (in press).

HENDRY, J.H., POTTEN, C.S., CHADWICK, C. & BIANCHI, M. (1982). Cell death (apoptosis) in the mouse small intestine after low doses: effects of dose-rate, 14.7 MeV neutrons, and 600 MeV (maximum energy) neutrons. *Int. J. Rad. Biol.*, **42**, (in press).

KERR, J.F.R. (1971). Shrinkage necrosis: a distinct mode of cellular death. *J. Pathol.*, **105**, 13.

KERR, J.F.R., WYLLIE, A.H. & CURRIE, A.R. (1972). Apoptosis: A basic biological phenomenon with wide-ranging implications in tissue kinetics. *Br. J. Cancer*, **26**, 239.

LU, C.C. & MEISTRICH, M.L. (1979). Cytotoxic effects of chemotherapeutic drugs on mouse testis cells. *Cancer Res.*, **39**, 3575.

MEISTRICH, M.L., FINCH, M., DA CUNHA, M.F., HACKER, U. & AU, W.W. (1982). Damaging effects of fourteen chemotherapeutic drugs on mouse testis cells. *Cancer Res.*, **42**, 122.

POTTEN, C.S. (1977). Extreme sensitivity of some intestinal crypt cells to X and $\gamma$-irradiation. *Nature*, **269**, 518.

POTTEN, C.S. (1980). Stem cells in small-intestinal crypts. In *Cell Proliferation in the gastrointestinal tract,* (Eds. Appleton *et al.*) Tunbridge Wells: Pitman Medical. p. 141.

POTTEN, C.S. (1983). Stem cells in gastrointestinal mucosa. *UCLA Symp.*, (in press).

POTTEN, C.S. & HENDRY, J.H. (1983). Stem cells in murine small intestine In *Stem Cells: Identification and Characterisation* (Ed. Potten), Edinburgh: Churchill-Livingstone. p. 155.

POTTEN, C.S., AL-BARWARI, S.E. & SEARLE, J. (1978). Differential radiation response amongst proliferating epithelial cells. *Cell Tissue Kinet.*, **11**, 149.

POTTEN, C.S., AL-BARWARI, S.E., HUME, W.J. & SEARLE, J. (1977). Circadian rhythms of presumptive stem cells in three different epithelia of the mouse. *Cell Tissue Kinet.*, **10**, 557.

POTTEN, C.S., CHWALINSKI, S., SWINDELL, R. & PALMER, M. (1982). The spatial organisation of the hierarchical proliferative cells of the crypts of the small intestine into clusters of "Synchronised" cells. *Cell Tissue Kinet.*, **15**, 351.

POTTEN, C.S., HENDRY, J.H., MOORE, J.V. & CHWALINSKI, S. (1983). Cytotoxic effects in gastrointestinal epithelium (as exemplified by small intestine) In *Cytotoxic Insult to Tissues: Effects on Cell Lineages,* (Eds. Potten & Hendry) Edinburgh: Churchill-Livingstone p. 105.

POTTEN, C.S., SCHOFIELD, R. & LAJTHA, L.G. (1979). A comparison of cell replacement in bone marrow, testis and three regions of surface epithelium. *Biochim. Biophys. Acta.*, **560**, 281.

POTTEN, C.S., HUME, W.J., REID, P. & CAIRNS, J. (1978). The segregation of DNA in epithelial stem cells. *Cell,* **15**, 899.

QUASTLER, H. & SHERMAN, F.G. (1959). Cell population kinetics in intestinal epithelium of the mouse. *Exp. Cell Res.*, **17**, 420.

SCHOFIELD, R. (1978). The relationship between the spleen colony-forming cell and the haemopoietic stem cell. *Blood Cells*, **4**, 7.

SEARLE, J., LAWSON, T.A., ABBOTT, P.J., HARMON, B. & KERR, J.F.R. (1975). An electron-microscope study of the mode of cell death induced by cancer-chemotherapeutic agents in populations of proliferating normal and neoplastic cells. *J. Pathol.*, **116**, 129.